# Intensity of Physical Activity in Young People: Focus on Emotional, Cognitive, and Healthy Lifestyle-Related Variables

**DOI:** 10.3390/bs14100935

**Published:** 2024-10-11

**Authors:** Adrián S. Elliott, Román D. Moreno-Fernández, Ana Cordellat-Marzal, Patricia Sampedro-Piquero

**Affiliations:** 1 Departamento de Psicología Biológica y de la Salud, Facultad de Psicología, Universidad Autónoma de Madrid, 28049 Madrid, Spain; adrian.sancheze@uam.es; 2 Facultad de Educación y Psicología, Universidad Francisco de Vitoria, 29223 Madrid, Spain; 3 Departamento de Educación Física y Deportiva, Universitat de València, 46010 Valencia, Spain; ana.cordellat@uv.es

**Keywords:** cognition, emotion, lifestyle, physical activity, youth

## Abstract

The college years represent a crucial developmental period in which unhealthy behaviors, including smoking, alcohol consumption, inadequate physical activity (PA), poor sleep quality, and unhealthy nutrition habits are often acquired, influencing the onset or exacerbation of pre-existing mental disturbances such as anxiety, depression, or difficulties in emotion regulation. Our aim was to analyze the effect of the intensity of physical activity (PA) on the emotional, behavioral, and cognitive variables in a sample of young people. We recruited 103 participants (19.85 ± 0.25) who completed several online questionnaires (IPAQ-SF, STAI-t, DERS, UPPS-P, ISP-20, and Mini-IPIP). Subsequently, face-to-face sessions were conducted to assess the cognitive variables and to collect more details about their lifestyle habits, including drug use, sleep quality, and leisure activities. Based on the IPAQ-SF results, we categorized the sample into three groups: light PA (n = 47), moderate PA (n = 29), and vigorous PA (n = 27). Vigorous PA group showed better emotional regulation, lower impulsivity, fewer prefrontal symptoms and unhealthy behaviors, better sleep quality, and more leisure activities. No significant cognitive differences were found. It seems that young people are a vulnerable group with emotional and impulsivity traits and engaging in intense PA appears to be a promising avenue for managing these symptoms. In conclusion, our study suggests that young people are a vulnerable group with emotional and impulsivity traits that may precipitate in future mental disorders. Nevertheless, engaging in PA, particularly at high intensity, appears to be a promising avenue for reducing and managing these symptoms during this critical period of life.

## 1. Introduction

The university years are a crucial period during which students transition from late adolescence to emerging adulthood [1]: a critical phase of personal development and in the building and reinforcement of self-esteem [2,3]. Epidemiological studies suggest that 12–50% of university students meet the criteria for one or more common mental disorders [4,5]. Studies have found that psychological disorders, such as anxiety or depression, are typically more prevalent among those aged 18–34 years than those aged 35–49 [6,7]. Additionally, mental disorders in early adulthood are associated with long-term adverse outcomes in later life, including persistent emotional and physical health problems [8] or relational disorders [9], where emotional dysfunction impairs the use of appropriate emotion regulation strategies [10]. However, research into emotional dysregulation among young people remains comparatively limited, especially in Spain, which may be due to the limited number of available measurement tools for this sample [11]. Another important trait associated with psychopathology and high-risk behavior among young people is impulsivity [12]. However, a few studies have examined the association between impulsivity, often evaluated using the UPPS-P questionnaire, and healthy behaviors during this period of life [13,14]. Thus, prevention or intervention strategies focused on reducing mental distress, improving behavior control, and encouraging healthy lifestyle habits are necessary, and the university environment could be an ideal scenario for this purpose.

It is well known that the difficulties and challenges of daily life may partially explain the onset or worsening of psychological distress and mental illness. This is a period when most students move to another city, state, or province, often leaving home for the first time and taking responsibility for themselves. In addition, university students face academic, social, and employment pressures, [15] and, with the increased time spent on studies and extracurricular activities, leisure time is often restricted [16]. Furthermore, unhealthy habits (including smoking, alcohol consumption, inadequate physical activity (PA), and poor nutritional choices) are often acquired in youth or early adulthood, and they are associated with a fourfold increase in total mortality among men and women in the general adult population [17]. However, despite the obvious consequences of such lifestyles, a high proportion of young people lead unhealthy lives, indulging in habits such as the avoidance of PA, an unsuitable diet, and a high intake of alcohol, tobacco, and illegal drugs [18].

Physical activity is considered ideal in promoting healthy brain functioning, cognition, and mental health throughout one’s life. PA has been broadly defined as any bodily movement produced by the contraction of skeletal muscle that increases energy expenditure above a basal level and involves detailed parameters such as intensity, type, and duration [19]. Among youth, PA has been shown to reduce substance abuse and symptoms of depression and anxiety while offering substantial benefits to physical and mental health [20]. These benefits can occur quickly, inducing positive moods and attitudes [21], or they may require weeks of consistent exercise before emerging [22]. Research into youth PA highlights the difficulty of initiating and maintaining this behavior, especially among university students who are often sedentary [23]. Studies have also found that those who initiate an exercise program do so sporadically or stop completely before reaping any of its many potential benefits [24,25]. By contrast, habitual exercise is associated with better emotional regulation strategies [26], greater inhibitory control [27], and greater resilience, strengthening self-regulation through top-down control of bottom-up processing [28]. Studies on young people suggest that high-intensity interval training (HIIT) could have more positive effects on cognitive performance and psychological outcomes [29], promoting higher levels of neurotrophic factors, such as brain-derived neurotrophic factors [30], synaptic plasticity [31], and increased cerebral blood flow [32]. Nonetheless, more studies on the intensity of PA among young people and its relationship with positive emotional and cognitive variables must be carried out.

Few studies have explored the effect of PA intensity on healthy behavior (drug use, sleep quality, leisure activities, etc.) and the psychological and cognitive variables among the young according to gender. These studies are even less common in Spain, where the limited evidence available suggests a high prevalence of alcohol and tobacco use and a low prevalence of PA, especially among women [33,34]. Unfortunately, approximately one third of Spanish university students reported suffering from a common mental disorder in the past year, and one third of those reported severe role impairment [35]. Women reported a higher prevalence of mood and anxiety disorders, while men showed higher levels of substance abuse [36]. Taking this into account, this study aimed to explore how regular PA at different intensities (light, moderate, and vigorous) can variously impact mental well-being and cognition, especially prefrontal-related functions and healthy lifestyle choices, among a sample of Spanish university students. We considered the obtained results to be relevant since, as we mentioned, there are few studies on the impact of exercise on lifestyle, cognition, and emotion, as well as potential gender-related differences, among young people in Spain. Likewise, understanding whether these changes may be greater or not depending on the intensity of physical activity can help promote specific physical activity guidelines for young people based on their physical and mental state. Finally, we hypothesized that certain mental difficulties are more prevalent among more sedentary participants, whereas moderate or vigorous PA will be related to mental well-being and other healthy behaviors, including sleep quality or reduced substance abuse. Moreover, we expected women to be more sedentary, with more frequent anxiety or emotional alterations in line with the findings of the existing literature. Thus, PA may constitute an interesting and non-stigmatizing activity to improve the well-being, habits, and competences of university students.

## 2. Method

### 2.1. Participants

This study used cross-sectional data from undergraduate students aged between 18 and 25 years. The recruitment followed the snow-ball sampling technique through verbal disclosure, e-mails, and notice boards at the Autonomous University of Madrid, Francisco de Vitoria, and the Complutense University of Madrid. The inclusion criteria were as follows: (1) aged 18–25 years; (2) college students; and (3) absence of diagnosed psychological disorders (anxiety, depression, substance use disorders, etc.). Volunteers were excluded if they presented severe difficulties in understanding the test instructions, altered consciousness or agitation, if they consumed prescription drugs affecting the central nervous system (mainly anxiolytics and antidepressants), and PA addiction, as revealed by the Spanish version of the Exercise Addiction Inventory (EAI—with a score above 12) [37]. The final sample was 103 Caucasians, of which 30 were male and 73 female, and they were aged 19.85 ± 0.25 and matched for education level. All volunteers included in this study signed an informed consent form accompanied by an informative note, and they created an alphanumeric code based on the first letter of their first name, the last letter of their first surname, followed by their date of birth (e.g., P02712) to guarantee privacy during the data processing and analysis phases. First, to collect information about PA performance, substance use, anxiety traits, emotional regulation, impulsivity, prefrontal symptoms, and personality traits, several online standardized questionnaires were administered (Qualtrics, Silver Lake Partners, 2002): the International Physical Activity Questionnaire-Short form (IPAQ-SF [38]; the Alcohol Use Disorder Identification Test (AUDIT, Spanish adaptation [39]; the Cannabis Abuse Screening Test (CAST) [40]; the State-Trait Anxiety Inventory (STAI-t) [41]; the Difficulties in Emotion Regulation Scale (DERS) Spanish adaptation [42]; the Impulsive Behavior Scale (UPPS-P) Spanish adaptation [43]; the Prefrontal Symptoms Inventory (PSI-20) [44]; and the Mini-IPIP [45]. Regarding physical activity, participants were asked in the IPAQ-SF to report the days, hours, and minutes spent last week on vigorous-intensity PA, moderate-intensity PA, and walking (which reflected light-intensity PA). IPAQ-SF is a widely used and valid 7-day recall measure for assessing various types of PA. The amount of PA was indicated by the metabolic equivalent (MET) of minutes per week, and this was calculated by multiplying the MET value (3.3 METs for walking, 4.0 METs for moderate-intensity PA, and 8.8 METs for vigorous-intensity PA) by the total minutes per week of each type of PA. Higher scores in MET-minutes per week indicated higher levels of PA [46]. Based on the classification criteria of the IPAQ-SF, we divided the sample into 3 groups according to their weekly PA intensity: light PA (mean MET = 436.58 + 48.47, n = 47), moderate PA (MET = 2062.69 + 75.31, n = 29), and vigorous PA (3994.04 + 172.29, n = 27).

This study was approved by the Ethics Committee of the Autonomous University of Madrid (code: CEI-122-2490) and conducted in accordance with the Ethical Principles for Medical Research Involving Human Subjects adopted in the Declaration of Helsinki by the World Medical Association (64th WMA General Assembly, Fortaleza, Brazil, October 2013), Recommendation No. R (97) 5 of the Committee of Ministers to Member States on the Protection of Medical Data (1997), and the Spanish Data Protection Act (Ley Orgánica 15/1999 de Protección de Datos, LOPD). All participants were duly informed about the study prior to their participation and provided their informed consent in writing.

### 2.2. Procedure

After completing the online questionnaires using the Qualtrics^®^ survey platform (Qualtrics, Silver Lake Partners, 2002), the participants were contacted by email and interviews were arranged to collect more details about sociodemographic variables, lifestyle behaviors (sleep: hours, difficulty in falling asleep, or sleep disorders; drug abuse: type of drug, onset, quantity, etc.; sedentary behavior: hours per day sitting, leisure activities, etc.) [47], and any diagnosed psychological disorders or medication. Participants also performed two prefrontal-dependent tests: the d2 test [48] to measure attentional and speed processing, and the Digit span test [49] to evaluate their working memory. Details of the testing procedures are as follows.

- *d*2 test [48]: In this test, participants must cross out any letter “d” with two marks around, above, or below in any order. There are also surrounding distractors that are similar to the target stimulus, e.g., a p with two marks or a d with one or three marks. The time limit per line of the test is 20 s.
- Digit span test [49]: This is used to measure the working memory’s number storage capacity. Participants hear a sequence of numbers and are required to recall the sequence correctly, with increasingly longer sequences being tested in each trial. Digit span tasks are given forward or backward, meaning that once the sequence is presented, the participant is asked to recall the sequence in either normal or reverse order.

Finally, the volunteers were instructed to record their daily steps using their watches or mobile devices with pedometers for 7 days, reporting the average number of steps taken at the end of the week. This helped to control the overestimated or biased score of the IPAQ-SF questionnaire, observing a positive Pearson correlation between both results (*r* = 0.85, *p* = 0.001). Table 1 shows the mean ± standard error of the mean (SEM) for each experimental condition (light PA; moderate PA; and vigorous PA).

### 2.3. Statistical Analysis

All statistical analyses were performed using SPSS 25 (IBM SPSS Statistics, Corporate headquarters, New Orchard Road, Armonk, New York 10504-1722, USA), and all *p* values were two-tailed; the level of significance was taken as *p* ≤ 0.05. Descriptive analyses (mean ± SEM) were performed on the demographic variables, the information related to drug use, and the online questionnaire and cognitive test results (see Table 1). When the assumption of normality was not met, MANOVA or non-parametric Kruskal–Wallis tests were performed for all measurements taken throughout this study (health behaviors and emotional and cognitive variables). Appropriate post hoc comparisons were conducted when significant differences were found. Additionally, a multiple linear regression analysis was performed in order to determine the relationship of physical exercise with the significant variables.

## 3. Results

**Figure 1:**
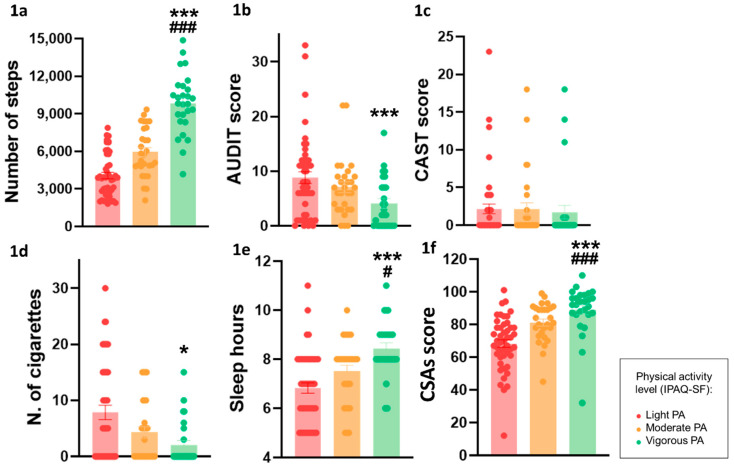
Differences among the light, moderate, and vigorous PA conditions in the average number of steps during a week (**1a**); alcohol (AUDIT) (**1b**), cannabis (CAST) (**1c**), and tobacco abuse (**1d**); sleep hours (**1e**); and CSAs questionnaire results (**1f**) (* vigorous vs. low; **#** vigorous vs. moderate). All data are the mean ± SEM, and statistically significant differences were considered when *p* ≤ 0.05 (*) (**#**) and *p* ≤ 0.005 (***) (**###**).

Statistically significant differences were found in the physical activity of the groups in terms of lifestyle variables, substance abuse, leisure activities, psychological traits, and prefrontal-dependent tests. First, and as an objective validation of the self-report questionnaire IPAQ-SF, the number of steps measured by their mobile or smart devices was significantly different in each group (*F* (_2,100_) = 72.21, *p* = 0.0001; Table 1). Tukey post hoc analysis revealed that these differences were level-dependent, with the vigorous PA group showing the highest levels, followed by the moderate PA group, and then, finally, the light PA group (with the lowest average number of steps) (Figure 1a).

### 3.1. Health- and Lifestyle-Related Variables

The results showed that alcohol use, assessed by AUDIT, was significantly less frequent in the vigorous PA group than in the light PA group (*F*(_2,100_) = 5.04, *p* = 0.008) (Figure 1b), whereas no differences were observed in cannabis use (CAST score, Figure 1c). Also, the tobacco consumption of the vigorous PA group was also significantly lower than the light PA group (*H*(_2_) = 9.05, *p* = 0.01) (Figure 1d). The average number of hours of sleep (*F*(_2,100_) = 12.44, *p* = 0.00001) was lower in the light PA group than in the moderate and vigorous group (Figure 1e). Regarding leisure activities, the vigorous PA group scored higher than both moderate and light groups (*F*(_2,100_) = 17.92, *p* = 0.00001) (Figure 1f) (see Table 1).

### 3.2. Psychological Variables

**Figure 2:**
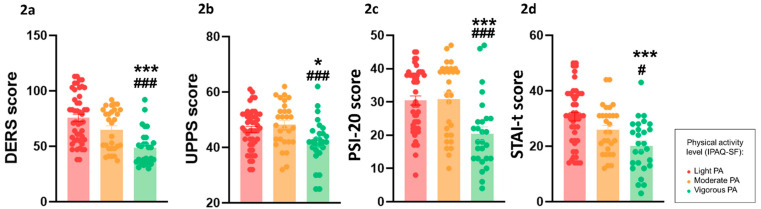
The differences among the light, moderate, and vigorous PA conditions in the total score of the DERS (**2a**), UPPS-P (**2b**), ISP-20 (**2c**), and STAI-t (**2d**) questionnaires, as well as in the sub-scales of the Extraversion and Conscientiousness of Mini-IPIP (* vigorous vs. low; **#** vigorous vs. moderate). All data are the mean ± SEM, and statistically significant differences were considered when *p* ≤ 0.05 (*) (**#**), *p* ≤ 0.005 (***) (**###**).

With regard to emotional regulation, the DERS scores (Figure 2a) were significantly higher in the light PA group (*H*_(2)_ = 24.83, *p* = 0.00001), with levels of difficulty receding as PA increased in the subscales: “Emotional neglect” (*F*_(2,100)_ = 5.96, *p* = 0.004), “Emotional confusion” *H*_(2)_ = 18.63, *p* = 0.0001), “Emotional interference” (*F*_(2,100)_ = 19.11, *p* = 0.00001), “Emotional dysregulation” (*H*_(2)_ = 26.69, *p* = 0.00001), and “Emotional rejection” (*F*_(2,100)_ = 4.86, *p* = 0.0097) (Table 1). The total score of the UPPS-P was also lower in the vigorous PA group (*F*_(2,100)_ = 4.82, *p* = 0.01) (Figure 2b), particularly in the subscales of “Negative urgency” (*F*_(2,100)_ = 4.27, *p* = 0.01) and “Lack of perseverance” (*F*_(2,100)_ = 5.90, *p* = 0.004). The total score of the PSI-20 was also lower in the vigorous PA group (Figure 2c) (*F*_(2,100)_ = 9.84, *p* = 0.0013), where the subscales of “Social behavior” (*F*_(2,100)_ = 3.99, *p* = 0.021) and “Behavioral control” (*F*_(2,100)_ = 10.76, *p* = 0.000001) were found to be significant (Table 1). Finally, we also found that the vigorous PA group displayed significantly less anxiety traits (STAI-t; F(2,100) = 10.13, *p* = 0.00001) than the other two groups (Figure 2d) (see Table 1).

### 3.3. Cognitive Variables

Finally, no statistically significant differences were found in either the attention task (d2) or the working memory test (Digit Span). The only tendency toward significance was in the attentional scores of correct responses (*F*(_2,100_) = 2.39, *p* = 0.09) and the CON index (*F*(_2,100_) = 2.37, *p* = 0.09), implying subtle differences in favor of the vigorous PA group with the highest scores (see Table 1).

### 3.4. Multiple Linear Regression

According to a linear regression analysis of the statistically significant variables above, we found a model with four predictors of the IPAQ score (*R*^2^Adjusted = 0.554, *F*(7,95) = 16.879, *p* < 0.0001=, with two negatives of DERS (*b* = −0.31, *p* = 0.016) and PSI-20 (*b* = −0.26, *p* = 0.013), and two positives of sleep hours (*b* = 0.34, *p <* 0.0001) and leisure activities (*b* = 0.42, *p <* 0.0001)). This result suggests that regular PA is, above all, associated with emotional- and health-related variables.

A supplementary material of this study (Table S1) shows the mean ± SEM by PA and gender. As this study did not focus on gender differences, these data were not included in the main manuscript.

## 4. Discussion

This study found that individuals engaging in vigorous PA demonstrated superior emotional regulation skills, enhanced impulse control, and fewer prefrontal symptoms in their daily routines compared to those in other groups. Moreover, the vigorous PA group exhibited a lower prevalence of unhealthy behaviors such as alcohol, cannabis, or tobacco use. Members of this group also reported better sleep quality and participated in a more diverse range of leisure activities compared to those with light or moderate PA. However, there were no notable differences among the groups in the evaluated cognitive functions, including attentional processes and working memory. Our linear regression analysis also revealed that self-reported PA intensity by IPAQ-SF was predicted by fewer emotional regulation difficulties, as well as prefrontal symptoms, and higher number of sleep hours and leisure activities. It constitutes a relevant finding highlighting how the higher the intensity of PA, the better emotional regulation and behavioral control, as well as the greater degree of healthier lifestyle habits in young people. This can also lead university campus contexts in which, through access to and promotion of PA, other types of psycho-affective and social aspects can be addressed with young people to improve their wellbeing.

### 4.1. A Higher Intensity of PA Was Related to Healthy Behaviors

We found that greater PA coincides with less consumption of substances such as alcohol or tobacco among university students. Studies in clinical samples have demonstrated the positive influence of physical activity, in conjunction with standard treatment, in reducing cravings, improving mood, and aiding in abstinence [50]. By contrast, data regarding youth populations with high-risk alcohol consumption are unclear, with contradictory findings in the literature on the relationship between levels of physical activity and alcohol consumption [51]. This effect is particularly pronounced in team and high-competition sports, where it is common to celebrate victories together and the team encourages and sometimes pressures players into alcohol consumption. Additionally, it appears that the association between moderate exercise and alcohol use is often strongest in men [52]. In our study, as shown in Table 1, most of the sample engaged in individual physical activity, primarily at the gym, while a small percentage in the vigorous group participated in team sports but not in a competitive setting. Furthermore, prior studies suggest that certain personality traits, such as extroversion, may serve as moderators in the relationship between exercise and alcohol [53]. To analyze these personality factors, we used the Mini-IPIP questionnaire, but we found no significant differences among the groups in this regard, nor in the rest of the sub-scales assessed. According to our results, highly extroverted individuals or those with pronounced levels of neuroticism were not found to be specifically at risk for increased alcohol abuse when participating in PA [54]. Additionally, it is important to note that some findings indicate a positive relationship between alcohol consumption and PA for men but not women [55], and the sample for this study consisted of 30 men and 73 women.

The average number of hours of sleep was lower in the light PA group than in the moderate and vigorous groups. Several studies have suggested that PA is an effective, non-pharmacological approach for improving sleep. In addition, PA is recommended as an alternative or complementary approach to existing therapies for sleep problems throughout life [56]. Inadequate sleep and irregular sleep–wake patterns observed in younger adolescents raises concerns about similar problems among university students. Poor quality of sleep is associated with reduced neurocognitive and academic performance, which persists from high school into the years in higher education [57]. Interestingly, other studies have also found that vigorous PA has a positive impact on sleep quality and is a predictor of better sleep than moderate PA [58]. Hence, greater effort must be made to promote PA among young people and to understand the impact of different intensities of PA.

Finally, the vigorous PA group not only spent their time performing PA, but also showed high scores in other healthy leisure activities, such as reading, listening to music, going to the cinema or theater, traveling, going out with friends, drawing, playing music, among other activities. Some studies have suggested that participation in leisure activities is a measure of the physical and mental health of an individual [59]. Thus, the evidence suggests that socially active leisure time during adolescence is a protective factor against the appearance of psychiatric disorders such as anxiety or substance abuse disorders in young adulthood [60].

### 4.2. Higher Intensity of PA Was Associated with Better Emotional and Impulse Control Skills

In line with previous research, the vigorous PA group exhibited better emotional regulation skills, specifically in the sub-scales of “Emotional neglect”, “Emotional confusion”, “Emotional interference”, “Emotional dysregulation”, and “Emotional rejection” compared to the other groups. Additionally, the sub-scales of PSI-20 “Social behavior” and “Behavioral control” also showed more positive outcomes with higher intensity PA. Finally, in relation to the UPPS-P questionnaire, the vigorous PA group showed lower scores in “Negative urgency” and “Lack of perseverance”, which may have been due to the ability of the vigorous PA group to implement positive re-interpretations and a fewer number of de-emphasizing reappraisals [61]. This positive appraisal style, which can be fostered, at least in part, by PA, was found to predict greater stress resilience [62]. Nonetheless, although numerous studies have found general positive associations between PA and mood, more research is needed to understand the nature of these effects. It has been suggested that PA can generate several physiological changes and mechanisms in the body, which, in turn, may lower stress levels or buffer the stress response protecting against the negative health effects of stress and improving mood and positive affect [63]. It has been also suggested that exercise relieves depressive and anxious symptoms by accumulating recurring mood enhancements. On the physiological level, regular PA is commonly associated with more flexible and adaptive stress responses of the cardiovascular and cortisol system, which may represent biological adaptations in terms of lower reactivity to or faster recovery from stressful events. Thus, it is expected that habitual PA should foster long-term mood elevation [26].

### 4.3. Prefrontal-Related Functions Were Not Affected by Different Intensities of PA

In contrast to previous research, we did not find a positive association between the intensity of PA and attention and working memory, despite there being evidence suggesting that sedentary habits are associated with poorer working memory while moderate and vigorous PA show the opposite [64]. Nonetheless, the exercise–cognition relationship is a complex phenomenon, and it is affected by several factors such as the modality, intensity, and duration of the exercise regime; age; physical fitness; and the cognitive domain being assessed [65]. These discrepant findings in the existing literature and our study may also reflect varying operational definitions of PA. Moreover, we should also consider that most studies have assessed the impact of PA on prefrontal-related functions immediately post-exercise while little is known about its long-term effectiveness or influence [66,67]. It is possible that many cognitive benefits of an intensive PA protocol are due to an increase in lactate and, consequently, the levels of the brain-derived neurotrophic factor (BDNF), which plays a key role in the promotion and maintenance of synaptic connections [68,69,70].

All in all, our study suggests that young people, given their vulnerability to emotional and impulsivity traits, may be at increased risk for developing future mental health disorders. However, engaging in physical activity, particularly at high intensity, shows promise as an effective approach for managing and reducing these symptoms during this critical developmental period, as well as for supporting improved emotional regulation, impulse control, sleep quality, and overall healthy lifestyle behaviors. Despite these promising results, it is important to note certain limitations in our study. Regarding the sample, although they are not very small, the use of a larger sample would increase the statistical evidence needed to draw stronger conclusions from the findings. Furthermore, the ratio of men to women was not homogenous in each group; thus, the findings of this study may be attributed to differential gender ratios in the different groups. First, our sample primarily consisted of women and Caucasian young adult university students; thus, the findings may not necessarily be generalizable to more diverse groups of young adults. Also, the participants were university students, and their data cannot be generalized to the general youth population. Second, the data were drawn from cross-sectional questionnaires, and a causal relationship between physical activity and the other analyzed variables could not be precisely determined. Additionally, the data from the self-report questionnaires may be overestimated or biased. Thus, for PA, we attempted to resolve these possible inaccuracies by including objective data from pedometers. Nevertheless, accelerometers may better reflect the actual level of the PA of participants. However, despite these limitations, we believe that the findings of this research constitute an advance for the development of prevention and intervention programs within the university context, where the aim is to build emotional competences, impulsivity control, and healthy lifestyles habits, which can help to optimize emotional, affective, academic, and social competences.

## Figures and Tables

**Table 1:** The emotional, cognitive, and behavioral results for each PA group.

	Light PA (n = 47)	Moderate PA (n = 29)	Vigorous PA (n = 27)	*p*
**Age**	20.19 ± 0.41	19.55 ± 0.33	19.59 ± 0.52	0.47
**AUDIT**	8.83 ± 1.08	7.07 ± 0.98	4.07 ± 0.89	0.008 **
**Age at onset for alcohol use**	15.23 ± 0.27	15.41 ± 0.37	16.04 ± 0.30	0.18
**CAST**	2.17 ± 0.65	2.14 ± 0.83	1.74 ± 0.90	0.92
**Cannabis use (YES %)**	23.4%	20.7%	11.1%	
**Nicotine (YES %)**	55.3%	48.3%	25.9%	
**Cigarettes/day**	7.83 ± 1.29	4.28 ± 1.02	2.04 ± 0.77	<0.001 *
**Sleep hours**	6.83 ± 0.21	7.52 ± 0.25	8.44 ± 0.22	<0.001 ***#
**Sleep difficulties**	51.1%	41.4%	11.1%	
**IPAQ-SF**	436.58 ± 48.47	2062.69 ± 75.31	3994.04 ± 172.29	<0.001 ***###
**EAI**	6.12 ± 0.45	7.87 ± 0.98	7.02 ± 1.03	0.69
**Type of PA (individual %)**	100%	100%	74.1%	
**Steps/week**	4075.06 ± 253.31	5938.72 ± 361.67	9832.81 ± 461.56	<0.001 ***###
**Sitting hours/day**				
**6–8 h**	29.8%	62.1%	22.2%	
**8–10 h**	59.6%	37.9%	70.4%	
**>10 h**	10.6%	-	7.4%	
**CSAs**	68.38 ± 2.38	81.21 ± 2.17	88.96 ± 2.88	<0.001 ***###
**STAI trait**	30.66 ± 1.53	25.93 ± 1.61	20.15 ± 1.81	<0.001 ***#
**DERS**	75.66 ± 3.27	65.07 ± 3.47	48.70 ± 3.12	<0.001 ***###
*Emotional neglect*	12.70 ± 0.59	10.69 ± 0.58	9.93 ± 0.62	0.004 ***###
*Emotional confusion*	7.57 ± 0.49	6.79 ± 0.36	4.67 ± 0.31	<0.001 ***###
*Emotional interference*	13.81 ± 0.54	12.03 ± 0.65	8.56 ± 0.61	<0.001 ***###
*Emotional dysregulation*	22.77 ± 1.32	18.79 ± 1.33	12.52 ± 0.90	<0.001 ***###
*Emotional rejection*	18.81 ± 1.06	16.76 ± 1.57	13.04 ± 1.43	0.007 **
**UPPS-P**	46.51 ± 1.06	48.24 ± 1.48	42.04 ± 1.59	0.01 *###
*Negative urgency*	9.64 ± 0.40	9.90 ± 0.42	8.04 ± 0.54	0.03 *
*Lack of perseverance*	8.49 ± 0.32	8.07 ± 0.51	6.52 ± 0.45	0.003 **
*Lack of premeditation*	7.72 ± 0.28	7.90 ± 0.40	7.33 ± 0.39	0.56
*Sensation seeking*	10.62 ± 0.44	11.86 ± 0.50	10.85 ± 0.65	0.21
*Positive urgency*	10.04 ± 0.45	10.52 ± 0.51	9.30 ± 0.62	0.32
**PSI-20**	30.43 ± 1.37	30.90 ± 2.03	20.48 ± 2.06	<0.001 ***###
*Emotional behavior*	6.32 ± 0.60	7.31 ± 0.71	4.81 ± 0.61	0.06
*Social behavior*	3.74 ± 0.47	5.24 ± 0.56	2.89 ± 0.62	0.02 *
*Behavioral control*	20.36 ± 0.92	18.34 ± 1.38	12.78 ± 1.35	<0.001 ***###
**Mini-IPIP**	65.43 ± 1.06	65.76 ± 1.01	67.26 ± 1.14	0.49
*Openness*	10.87 ± 0.23	11.17 ± 0.33	11.63 ± 0.41	0.23
*Conscientiousness*	13.85 ± 0.36	12.62 ± 0.53	12.37 ± 0.55	0.11
*Extraversion*	13.34 ± 0.52	14.83 ± 0.55	16.22 ± 0.49	0.08
*Agreeableness*	15.34 ± 0.38	14.62 ± 0.55	15.52 ± 0.44	0.39
*Neuroticism*	12.02 ± 0.42	12.52 ± 0.43	11.19 ± 0.41	0.15
**COGNITIVE VARIABLES**				
*d2–correct*	158.62 ± 6.68	158.14 ± 7.65	178.59 ± 6.01	0.09
*d2–omissions*	21.87 ± 3.07	21.03 ± 4.10	13.41 ± 2.73	0.19
*d2–commissions*	2.66 ± 0.70	4.17 ± 1.17	1.30 ± 0.58	0.10
*d2–TOT*	414.13 ± 12.61	407.86 ± 15.14	436.52 ± 11.96	0.36
*d2–VAR*	12.13 ± 0.61	12.10 ± 1.10	11.41 ± 0.78	0.80
*d2–CON*	155.98 ± 7.07	153.93 ± 8.37	176.59 ± 6.41	0.09
*Forward digits*	9.36 ± 0.38	9.69 ± 0.42	10.22 ± 0.36	0.32
*Backward digits*	7.19 ± 0.39	7.38 ± 0.43	7.56 ± 0.39	0.82
*Total digits*	16.47 ± 0.74	17.21 ± 0.82	17.78 ± 0.70	0.48

Values are the means ± SEM or percentages. (* vigorous vs. low; # vigorous vs. moderate). All data are the mean + SEM, and statistically significant differences were considered when *p* < 0.05 (*) (#), *p* < 0.01 (**), and *p* < 0.005 (***) (###). AUDIT: Alcohol Use Disorders Identification Test. CAST: Cannabis Abuse Screening Test. IPAQ-SF: International Physical Activity Questionnaire—Short form. EAI: Exercise Addiction Inventory. CSAs: Cognitively Stimulating Activities scale. STAI trait: State-Trait Anxiety Inventory. DERS: Difficulties in Emotion Regulation Scale. UPPS-P: Impulsive Behavior Scale. PSI-20: The Prefrontal Symptoms Inventory—20. Mini-IPIP: Mini-International Personality Item Pool.

## Data Availability

Data are available on request from the corresponding authors.

## Supplementary Materials

The following supporting information can be downloaded at: https://www.mdpi.com/article/10.3390/bs14100935/s1, Table S1. Emotional, cognitive and behavioral results for each group and sex.
