# Supplementary material for: Intensity of Physical Activity in Young People: Focus on Emotional, Cognitive, and Healthy Lifestyle-Related Variables
Source: Behav Sci (Basel). 2024 Oct 11;14(10):935. doi: 10.3390/bs14100935 (PMC11504200; doi:10.3390/bs14100935)
Supplement: Supplementary file 1 [file behavsci-14-00935-s001.zip › behavsci-3236976-supplementary.pdf]

Table S1. Emotional, cognitive and behavioral results for each group and sex.

	Light PA		Moderate PA		Vigorous PA	
	**Boys (n=13)**	**Girls (n=34)**	**Boys (n=10)**	**Girls (n=19)**	**Boys (n=7)**	**Girls (n=20)**
**Age**	22.31±0.90	19.38±0.37	21.10±0.62	18.74±0.23	18.86±0.51	19.85±0.68
**AUDIT**	15.77±2.30	6.18±0.86	8.90±2.49	6.10±0.70	3.29±1.41	4.35±1.10
**Age onset alcohol**	13.62±0.29	15.85±0.29	16.20±0.77			
**CAST**	5.46±1.81	0.91±0.41	3.70±1.55	1.32±0.95	2.14±1.98	1.60±1.02
**Cannabis use (YES % >4 cast)**	61.5%	8.8%	40%	10.5%	14.3%	10%
**Nicotine (YES %)**	69.2%	50%	50%	47.4%	28.6%	25%
**Cigarettes/day**	12.92±2.92	5.88±1.26	4±1.63	4.42±0.32	2.86±2.14	1.75±0.75
**Sleep hours**	7±0.36	6.76±0.26	7.40±0.27	7.58±0.35	8.86±0.50	8.30±0.24
**Sleep difficulties (YES%)**	46.2%	52.9%	50%	36.8%	14.3%	10%
**IPAQ-SF**	492.42±88.04	380.74±58.73	2046.20±124.87	2071.37±96.71	3913.14±396.11	4022.35±193.22
**Type of PA (individual %)**	100%	100%	100%	100%	100%	65%
**Steps/week**	3744.46±504.42	4201.47±294.13	6041.70±745.33	5884.53±404.85	10495.14±888.62	9601±543.48
**Sitting hours/day**						
**6-8h**	61.5%	17.6%	60%	63.2%	85.7%	30%
**8-10h**	38.5%	67.6%	40%	36.8%	14.3%	65%
**>10h**	-	14.7%	-	-	-	5%
**CSAs**	60.69±4.99	71.32±2.55	76.70±4.47	83.58±2.25	94.14±1.18	87.15±3.81
**STAI trait**	23.54±2.56	33.38±1.66	28.30±3.31	24.68±1.74	12.14±2.99	22.95±1.86
**DERS**	64.54±3.99	79.91±4.06	69±4.77	63±4.68	44.57±6.80	50.15±3.53
Emotional neglect	12.38±0.91	12.82±0.74	12.20±0.93	9.89±0.68	9.29±1.55	10.15±0.66
Emotional confusion	6.54±0.69	7.97±0.62	7.10±0.60	6.63±0.46	4.14±0.55	4.85±0.37
Emotional interference	11.23±0.88	14.79±0.59	12.50±0.34	11.79±0.98	9.43±0.92	8.25±0.76
Emotional dysregulation	18.77±2.29	24.29±1.54	19.20±2.08	18.58±1.75	10.14±0.77	13.35±1.14
Emotional rejection	15.62±1.72	20.03±1.26	18±3.09	16.11±1.82	11.57±3.92	13.55±1.43
**UPPS-P**	52.08±0.92	44.38±1.25	35.40±2.52	46.11±1.49	40.71±4.16	42.50±1.65
Negative urgency	9.54±0.45	9.68±0.53	10.40±0.75	9.63±0.50	6.57±1.23	8.55±0.55
Lack of perseverance	8.92±0.87	8.32±0.30	9.90±1.10	7.11±0.40	7±0.76	6.35±0.55
Lack of premeditation	8.38±0.43	7.47±0.34	8.50±0.67	7.58±0.50	6.86±1.01	7.50±0.41
Sensation seeking	12.77±0.52	9.79±0.51	11.70±0.99	11.95±0.57	11.43±1.49	10.65±0.72
Positive urgency	12.46±0.39	9.12±0.53	11.80±0.81	9.84±0.58	8.86±1.47	9.45±0.68
**PSI-20**	29.15±2.62	30.91±1.62	35.40±2.50	28.53±2.69	19±5.31	21±2.16
Emotional behavior	3.77±0.54	7.29±0.75	7.60±1.11	7.16±0.93	2.29±0.89	5.70±0.66
Social behavior	5.31±1.12	3.15±0.47	6.6±0.91	4.53±0.67	3.86±1.77	2.55±0.59
Behavioral control	20.07±2.15	20.47±0.99	21.20±1.66	16.84±1.85	12.86±3.28	12.75±1.47
**Mini-IPIP**	63.54±2.14	66.15±1.21	63.60±1.67	66.89±1.22	62.29±2.99	69±0.89
Openness	11.77±0.30	10.53±0.28	11.50±0.52	11±0.43	12.71±1.04	11.25±0.43
Conscientiousness	12.54±0.73	14.35±0.39	12.30±0.83	12.79±0.69	10.86±0.88	12.90±0.65
Extraversion	14.39±0.80	12.94±0.65	14.30±0.91	15.11±0.70	16.14±1.26	16.25±0.52
Agreeableness	14.31±0.84	15.74±0.40	12.80±1.06	15.58±0.51	14.14±0.94	16±0.47
Neuroticism	10.54±0.48	12.59±0.53	12.70±0.79	12.42±0.52	8.43±0.30	12.15±0.33

COGNITIVE VARIABLES

d2 – correct	146.92±13.45	163.09±7.66	138.30±10.01	168.58±9.75	190.86±13.24	174.30±6.61
d2 – omissions	25.92±7.63	20.32±3.13	20.50±8.99	21.31±4.31	11.71±2.30	14±3.62
d2 – commissions	3±1.06	2.53±0.88	4.80±2.15	3.84±1.42	-	1.75±0.76
d2 – TOT	385.85±23.10	424.94±14.81	359.50±18.58	433.32±18.68	469±28.03	425.15±12.30
d2 – VAR	12.92±1.57	11.82±0.61	9±0.68	13.74±1.52	11.29±0.64	11.45±1.03
d2 – CON	143.92±14.14	160.59±8.13	133.50±11.53	164.68±10.62	188.14±14	172.55±7.14
Forward digits	7.31±0.51	10.14±0.42	9.70±0.84	9.68±0.48	10.29±0.52	10.20±0.45
Backward digits	5.07±0.29	8±0.45	8.10±0.91	7±0.43	7.43±0.37	7.60±0.52
Total digits	12.08±0.51	18.45±0.84	18±1.83	16.79±0.83	17.71±0.68	17.80±0.93

Values are means ± SEM or percentages. AUDIT: Alcohol Use Disorders Identification Test. CAST: Cannabis Abuse Screening Test. IPAQ-SF: International Physical Activity Questionnaire – Short form. CSAs: cognitively stimulating activities scale. STAI trait: State-Trait Anxiety Inventory. DERS: Difficulties in Emotion Regulation Scale. UPPS-P: Impulsive Behavior Scale. PSI-20: The Prefrontal Symptoms Inventory – 20. Mini-IPIP: Mini-International Personality Item Pool.
